# Non-functional alternative splicing caused by a Latino pathogenic variant in a case of PMM2-CDG

**DOI:** 10.1016/j.ymgmr.2021.100781

**Published:** 2021-07-02

**Authors:** C.A. González-Domínguez, C.E. Villarroel, M. Rodríguez-Morales, S. Manrique-Hernández, A. González-Jaimes, F. Olvera-Rodriguez, K. Beutelspacher, C. Molina-Garay, K. Carrillo-Sánchez, L.L. Flores-Lagunes, M. Jiménez-Olivares, A. Muñoz-Rivas, M.E. Cruz-Muñoz, H.M. Mora-Montes, R. Salinas-Marín, C. Alaez-Verson, I. Martínez-Duncker

**Affiliations:** a Laboratorio de Glicobiología Humana y Diagnóstico Molecular, Centro de Investigación en Dinámica Celular, Instituto de Investigación en Ciencias Básicas y Aplicadas, Universidad Autónoma del Estado de Morelos, Cuernavaca 62209, Mexico; b Instituto de Biotecnología, Universidad Nacional Autónoma de México, Cuernavaca 62210, Mexico; c Departamento de Genética, Instituto Nacional de Pediatría, Ciudad de México 04530, Mexico; d Laboratorio de Diagnóstico Genómico, Instituto Nacional de Medicina Genómica, Secretaría de Salud, Ciudad de México 14610, Mexico; e Laboratorio de Inmunología Molecular, Facultad de Medicina, Universidad Autónoma del Estado de Morelos, Cuernavaca 62209, Mexico; f Departamento de Biología, División de Ciencias Naturales y Exactas, Campus Guanajuato, Universidad de Guanajuato, Guanajuato 36050, Mexico

## Abstract

We report on a Mexican mestizo with a multisystemic syndrome including neurological involvement and a type I serum transferrin isoelectric focusing (Tf IEF) pattern. Diagnosis of PMM2-CDG was obtained by clinical exome sequencing (CES) that revealed compound heterozygous variants in *PMM2,* the encoding gene for the phosphomannomutase 2 (PMM2). This enzyme catalyzes the conversion of mannose-6-P to mannose-1-P required for the synthesis of GDP-Man and Dol-P-Man, donor substrates for glycosylation reactions. The identified variants were c.422G>A (R141H) and c.178G>T, the former being the most frequent *PMM2* pathogenic mutation and the latter a previously uncharacterized variant restricted to the Latino population with conflicting interpretations of pathogenicity and that we here report causes leaky non-functional alternative splicing (p.V60Cfs*3).

## 1 Introduction

Congenital Disorders of Glycosylation (CDG) are a heterogeneous group of nearly 137 genetic diseases due to defective glycoprotein and glycolipid glycan synthesis and attachment [24] Glycoprotein glycosylation defects can be divided into *N*-glycosylation defects, *O*-glycosylation defects and C-mannosylation defects. Screening for *N*-glycosylation defects mostly occurs by serum transferrin isoelectric focusing (Tf IEF) [21], but not all CDGs show abnormal transferrin, and some affected individuals normalize glycosylation, in time, without clinical improvement [10].

The most frequent *N*-glycosylation disorder is PMM2-CDG (OMIM 212065), an autosomal recessive multisystemic syndrome [30]. PMM2-CDG is a disorder of protein *N*-glycosylation characterized by genetic defects leading to deficiency/dysfunction of PMM2, the enzyme responsible for the conversion of mannose-6-phosphate into mannose-1-phosphate [11,22]. Mannose-1-phosphate is a precursor of guanosine diphosphate mannose (GDP-Man) and dolichol-P-mannose (Dol-P-Man). Deficiency of GDP-Man and Dol-P-Man causes hypoglycosylation of numerous glycoproteins, including serum and membrane glycoproteins. This results in multi-organ involvement, whereas disease severity and course variability are not fully understood [1].

We recently described the first case of PMM2-CDG in a Mexican mestizo patient [13], recommending increased awareness of CDG and particularly of PMM2-CDG because of the significant prevalence of pathogenic variants known for this gene. Underdiagnosis of CDGs is likely important, particularly in Latin America where few cases have been reported [3,4,7,13,20].

Here, we describe the second case of PMM2-CDG in a Mexican mestizo involving compound heterozygous pathogenic variants, demonstrating that one of them (c.178G>T), previously uncharacterized, disrupts the canonical donor splice site and causes a non-functional alternative splicing that originates a frameshift and premature stop codon (p.V60Cfs*3).

## 2 Clinical report

The patient is a 4 year and 3-month-old boy, the firstborn of a young, healthy, and non-consanguineous couple from Mexico City. His family history was unremarkable. He was obtained at term by C-section due to oligohydramnios after a previously uneventful pregnancy. His birth weight was 3070 g (z-score = −0.64), length 51 cm (z-score = −0.22), and his Apgar score was 9 at 5 min. Since birth, a right preauricular appendix was identified, and a subsequent renal ultrasound and hearing and neonatal screenings were all normal. From 4 months of age, hypotonia, psychomotor retardation and esotropia were evident, for which he was evaluated with a cranial CT scan that pointed out apparent cerebellar agenesis and prompted his referral to the National Institute of Pediatrics (México City). He was assessed in the Genetics Clinic at the age of 21 months. His physical examination confirmed central hypotonia and global neurodevelopmental delay. Microcephaly (z-score of head circumference = −3.02, z-score of height = −1.73) and other facial dysmorphisms with telecanthus, depressed nasal bridge, and widely spaced teeth with fusion of right upper incisors were also found. No evidence of abnormal fat depositions or inverted nipples was found.

MRI scan confirmed the cerebellar malformation showing a remarkably small size of the cerebellar vermis and the hemispheres. Due to these findings, a blood karyotype was performed, with a 46, XY normal result. Currently, he is under a physical therapy program and neurological surveillance. He started walking with support and has language use with less than ten words. His routine metabolic panel and blood count have not revealed any problem with kidney or liver function.

## 3 Materials and methods

### 3.1 Informed consent

Informed consent was obtained from both parents to perform a skin biopsy, fibroblast cultures and all required research to obtain a molecular diagnosis and to publish other data on the patient.

### 3.2 Transferrin isoelectric focusing (Tf IEF)

Serum from the patient (100 μL) was iron saturated at room temperature for 1 h with 5 μL of 0.5 M NaHCO_3_ and 5 μL of 20 mM FeCl_3_. One microliter of 10-fold-diluted serum was spotted on polyacrylamide gels (*T* = 5%, C = 3%) containing 5.7% ampholytes (pH 5–7). After electrophoresis, the gel was covered with 100 μL of an in-house rabbit anti-transferrin serum for 30 min at 4 °C. The gel was washed overnight with physiological saline, fixed, stained with Coomassie Brilliant Blue R-250, destained, dried, and photographed.

### 3.3 Cell culture

From a skin biopsy obtained from the patient, primary culture of fibroblasts was obtained in AmnioMAX™ C-100 Basal Medium (Gibco® by life technologies ™) supplemented with 15% AmnioMAX™ C-100 Supplement (Gibco® by life technologies ™) and 1% penicillin/streptomycin antibiotic (Gibco® by life technologies ™). Fibroblast cultures were maintained at 37 °C in a humidified atmosphere containing 5% CO_2_. Fibroblasts were further processed to obtain genetic material.

### 3.4 Clinical exome sequencing (CES)

Genomic DNA (gDNA) was extracted from fibroblasts using Maxwell ® 16 Blood DNA Purification Kit (Promega, Madison, WI, USA). The purity and concentration of the DNA samples were measured using NanoDrop 1000 spectrophotometer (Thermo Fisher Scientific, Waltham, MA, USA) and Qubit fluorometer (Thermo Fisher Scientific, Waltham, MA, USA). Library preparation was performed using the reagents provided in the Clinical Exome sequencing panel kit, version 2 (Sophia Genetics SA, Saint Sulpice, Switzerland), according to the manufacturer's protocol. Sequencing was performed on NextSeq Instrument (illumina San Diego, CA). Sequencing data analysis and variant annotation were performed with the Sophia DDM® software version 5.9.1.1 (Sophia Genetics SA, Saint Sulpice, Switzerland). A bioinformatic filter was constructed, including all the genes previously reported to be related to CDG.

### 3.5 Predictions of the pathogenicity of the variants

The HSFpro software (Genomnis) [9] was used to predict the effect of mutations on splicing. All predictions were made with the DYSF transcript ENST00000268261.

### 3.6 Sanger sequencing

The cDNA-based polymerase chain reaction (PCR) product corresponding to the coding sequence of *PMM2* was obtained using forward primer PMM2s 5′-TGCCAACGTGTCTTGTAAGG-3′ and reverse primer PMM2as 5′-GGAAGTTTCTGGCACTGGAG-3′ [32]. Sequencing of PCR products was performed by an ABI Prism 3130xl autoanalyzer (Applied Biosystems, Foster City, CA) and results were visualized using SnapGene Viewer 2.2.2 (GSL Biotech LLC, Chicago, IL, USA).

### 3.7 Allele specific PCR for the c.178G>T mutation

Primer PMM2mut-S 5′-GCAGGAGCAACTGGGAAATGAT**T**-3′ and PMM2-mutAS 5′-GGCCTC ACCCAGATGACTTTG-3′ were designed to detect the presence of the *PMM2* c.178G>T mutation. PCR with both primers results in a 127 bp amplicon.

## 4 Results

**Fig. 1:**
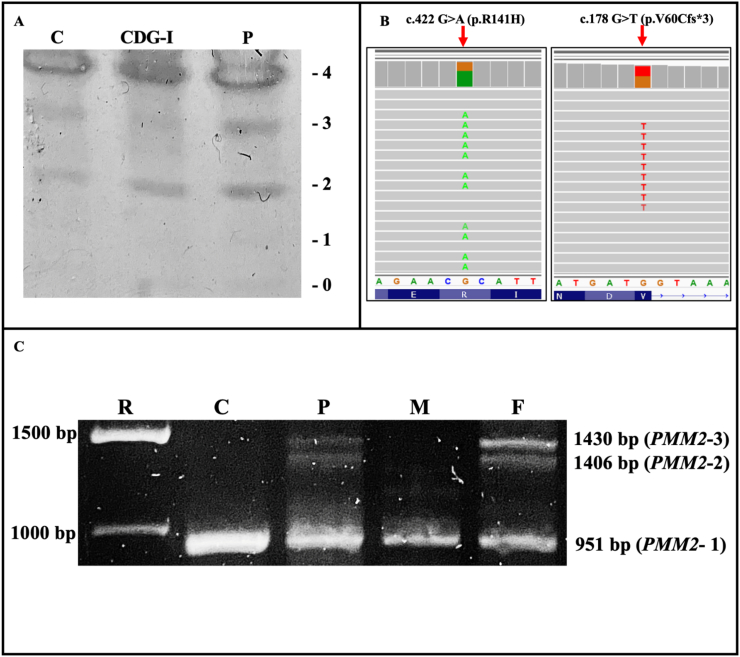
A. Serum Tf IEF showing an abnormal type I CDG profile; C is the sample from a healthy individual; CDG-I sample with abnormal type I profile; P is the serum sample from the patient. B. IGV (Integrative Genomics View) visualization of the c.422G>A and c.178G>T mutations. C. PCR products of *PMM2* cDNA amplification showing amplicons *PMM2*–1, *PMM2*–2 and *PMM2*–3 in the patient (P) and his father (F) while in a healthy individual (C) and his mother (M) only *PMM2*–1 was identified; R, ruler.

The patient's serum Tf IEF showed a mildly abnormal type I pattern with increased disialotransferrin with respect to the healthy control (Fig. 1A). In view of this result, gDNA obtained from fibroblasts were sent to CES and two known variants in *PMM2* were identified (Fig. 1B): one pathogenic missense mutation in exon 5 (c.422G>A, p.R141H) ClinVar 7706, the most prevalent mutation in PMM2-CDG and c.178G>T. The latter is located in exon 2 and reported in ClinVar 530390 with conflicting interpretations of pathogenicity (pathogenic and uncertain significance) and theoretically annotated to cause a p.V60L substitution.

According to the ACMG/AMP guidelines [29] the c.178G>T mutation is likely pathogenic. Valine60 is a highly conserved amino acid and the change to leucine involves a small physicochemical difference that is predicted to be pathogenic by DANN, FATHMM-MKL, M-CAP and MutationTaster but tolerated by BayesDel_addAF and EIGEN algorithms.

Because the replacement of c.178G>T modifies the last nucleotide of exon 2, it suggests that the pathogenicity of this variant could abrogate the donor splice site and induce aberrant alternative splicing. Using the Human Splicing Finder prediction (HSFPro, Genomnis) it was found that this mutation altered significantly altered the WT donor site, most probably affecting splicing with the following values [WT-Mut %variation] (HSF Donor site (matrix GT) 84.54 > 74.03 (−12.43%) and MaxEnt Donor site 6.41 > −0.21 (−103.28%)).

To further investigate this, the patient's and parents' cDNA was synthesized and *PMM2* was PCR amplified to detect alternative splicing isoforms. The human *PMM2* coding region spans over 738 bp and codes for a 246 aminoacid protein. Amplification of *PMM2* with primers PMM2s and PMM2as encompasses the coding sequence plus short stretches from the 5′- and 3´-UTR for a predicted amplicon of 951 bp. PCR product analysis in agarose gel showed that the healthy control and the mother had one single *PMM2* amplicon with the expected *wt* size (*PMM2*–1). Nonetheless, the patient and the father presented two additional amplicons with approximately 450 bp increase in size, termed *PMM2*–2 and *PMM2*–3, respectively (Fig. 1C), suggesting alternative splicing.

**Fig. 2:**
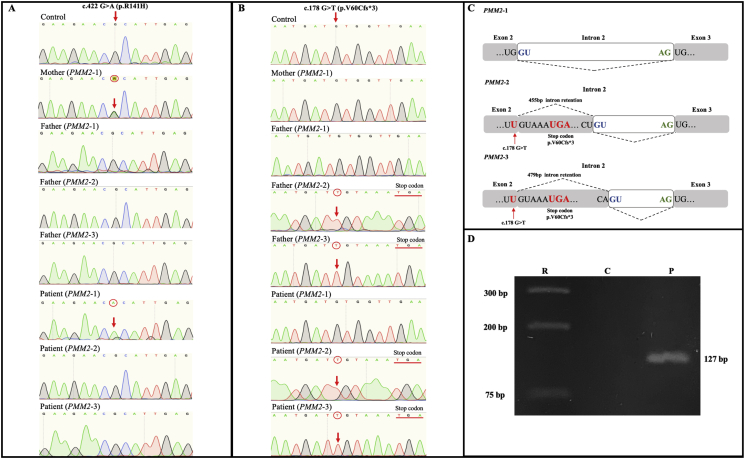
A. Sanger sequencing chromatogram showing the c.422G>A mutation in *PMM2–*1 from mother and patient, whereas this mutation is not present in a healthy individual or isoforms *PMM2*–2 and *PMM2*–3 from the father and patient. B. Sanger sequencing chromatogram showing the c.178G>T mutation in *PMM2*–2 and *PMM2*–3 from the father and patient while the absence of this mutation is shown in a healthy individual, and *PMM2*–1 from the mother and patient. C. Alternative splicing induced by the c.178G>T mutation produces partial intron 2 retention through the activation of two cryptic intronic donor splice sites, causing in both cases, an ORF change and premature stop codon. D. Allele-specific PCR detection of the c.178G>T mutation using the wt *PMM2* (C) or the patient's (P) *PMM2*–1 amplicon as template.

Sanger sequencing of the patient's *PMM2*–1 revealed a transcript with constitutive splicing that exclusively presented the c.422G>A (p.R141H) mutation, but not the c.178G>T variant (Fig. 2A), indicating that it was derived from the mother's mutated allele. On the other hand, the patient's and father's alternatively spliced *PMM2*–2 and *PMM2*–3 exclusively showed the c.178G>T, indicating paternal allele inheritance (Fig. 2B). The mother's *PMM2*–1 did not show the c.178G>T variant.

Although Sanger sequencing of *PMM2*–1 only detected the c.422G>A (R141H) mutation, it is known that this type of sequencing may not detect minor isoforms. Because of this, we designed an allele-specific PCR to detect the c.178G>T mutation. The *wt* PMM2 amplicon from the control and the patient's *PMM2*–1 were used as template. An allele-specific-PCR product was detected only in the patient (Fig. 2D) confirming the presence of transcripts carrying the c.178G>T mutation in the patient but not in the control. This indicates that the c.178G>T is a leaky splice mutation that allows minor constitutive splicing coding for PMM2 protein variants with the p.V60L mutation.

Sanger sequencing of *PMM2*–2 and *PMM2*–3 confirmed as predicted by the HSFPro software that the c.178 G>T variant disrupts the normal splicing donor site in the exon 2/intron 2 junction (UG/GU to UU/GU), activating two intronic cryptic donor splice sites and causing partial retention of intron 2. The *PMM2*–2 and *PMM2*–3 are characterized by isoforms with a 455 bp and 479 bp partial intron 2 retention, respectively. In both cases, abrogation of the *wt* donor splice site and partial intron retention leads to a frameshift that causes a premature stop codon +6 bp from the mutation (Fig. 2C).

## 5 Discussion

We recently reported the first Mexican mestizo with PMM2-CDG that also showed a compound heterozygous combination involving the c.422G>A (p.R141H) mutation [13], the most frequent *PMM2* mutation reported worldwide and consistent with a recent study in a cohort of 805 Mexican individuals being studied for infertility and that were genetically screened and where *PMM2* was one of the top 10 genes carrying pathogenic variants [13,16]. The c.178G>T mutation reported in the present work was not identified in this cohort (personal communication).

This report presents a patient with a mild clinical phenotype and IEF type I pattern consistent with CDG, noting that no inverted nipples or abnormal fat pads were observed as frequently occurs in PMM2-CDG. Therefore, CES was performed and two *PMM2* variants were found: the well-known c.422G>A (p.R141H) pathogenic variant and the c.178G>T variant that is annotated as likely pathogenic, according to ACMG/AMP guidelines, but with missing data on functional characterization or published reports. Interestingly, according to GnomAD exomes and genomes database [18], the c.178G>T variant is restricted to Latino ethnic origin with a frequency of 0.000174 or 1 in 5751 (gnomAD Exomes Version:2.1.1, as of April 2021).

To further characterize the mechanism of pathogenicity of the c.178G>T variant and in view that it is located −1 from the donor splicing site in exon 2, we considered that there was a high probability that this variant caused alternative splicing. This was first evidenced in gel-electrophoresis of *PMM2* cDNA PCR amplicons of the patient and the father. Confirmation was obtained by Sanger sequencing, demonstrating that alternatively spliced isoforms *PMM2*–2 and *PMM2*–3 were derived from the paternal allele as they exclusively carry the c.178G>T mutation.

Sequencing of *PMM2*–2 and *PMM2*–3 further revealed that the c.178G>T variant disrupts the normal donor splice site and results in partial intron 2 retention due to activation of two cryptic donor splice sites, causing ORF change and the generation of a premature stop codon p.V60Cfs*3 in both cases (Fig. 2C). We consider that the resulting protein would have no activity as amino acids R123, R134 and R141 needed for donor substrate binding of GDP-mannose would be affected [2]. Most probably, both alternatively spliced transcripts produced by the c.178G>T mutation may be degraded by the nonsense-mediated RNA decay pathway that selectively degrades mRNA harboring premature translation-termination codons, reducing the expression of truncated proteins [12]. Only two other mutations, c.178+1G>A (ClinVar 929132) and c.178+2T>G (ClinVar 553770), potentially affecting the same canonical donor splicing site, have been reported but without studies evaluating alternative splicing consequences.

An important finding was that although Sanger sequencing did not detect the c.178G>T mutation in the patient's*PMM2*–1 amplicons, the use of an allele-specific PCR allowed the identification of isoforms with constitutive splicing that present this mutation (Fig. 2D). The cause of this discrepancy is explained because of minor expression of these transcripts is below the sensitivity threshold of Sanger sequencing (limit of detection 15–20%). This is relevant because the c.422G>A (R141H) mutant is almost inactive and no patient has been reported with this mutation in a homozygous trait, always requiring of a hypomorphic mutant [27]. We therefore propose that the minor PMM2 p.V60L coded by the c.178G>T mutation in constitutively spliced isoforms is a fully or partially active variant that would allow residual PMM2 activity in this patient, particularly given his mild phenotype. Further experiments are required to characterize the functional impact of this mutation on enzyme activity.

Given these results, we conclude that the c.178G>T mutation causes the leaky p.V60L and non-functional alternative splicing (p.V60Cfs*3) and that alongside the well-characterized c.422G>A (p.R141H) mutation genetically confirms a PMM2-CDG diagnosis. In most disease-related genes, mutations affecting splicing are not fully characterized because mutation screening is restricted to gDNA. For example, of the 28 pathogenic or likely-pathogenic variants of *PMM2* affecting splicing (Table 1), only 9 have been functionally characterized regarding alternative splicing. In our experience, amplification of cDNA transcripts of *PMM2*, or any other gene, is an invaluable tool to demonstrate non-functional alternative splicing and better understand the pathogenic mechanisms it involves.

**Table 1:** Reported variants of PMM2 affecting splicing sites (NM_000303.3).

Mutation	Splicing site affected	Splicing data	Clinical significance reported in ClinVar	Reference
c.66+1G>T	Donor, intron 1	No	Pathogenic Likely pathogenic	Monin et al. [23]
c.66+1del	Donor, intron 1	No	Likely pathogenic	
c.67-2A>T	Acceptor, intron 1	No	Likely pathogenic	
c.178G>T	Donor, exon 2	Partial intron retention	Pathogenic VUS	Present work
c.178+1G>A	Donor, intron 2	No	Likely pathogenic	
c.178+2T>G	Donor, intron 2	No	Likely pathogenic	
c.179-1G>T	Acceptor, intron 2	No	Likely pathogenic	
c.179 -25A>G	Branch site sequence, intron 2	Exon 3 skipping	N/A	Vuillaumier-Barrot et al. [37]
c.255G>A	Donor, exon 3	Exon 3 skipping	Likely pathogenic	le Bizec et al. [5]
c.255+1G>A	Donor, intron 3	No	Pathogenic Likely pathogenic	Cabezas et al. [8]; Grünewald et al. [15]; Jones et al. [17]; Pérez-Dueñas et al. [26]
c.255+2T>C	Donor, intron 3	No	Pathogenic	Briones et al. [6]; Grünewald et al. [15]; le Bizec et al. [5]; Monin et al. [23]; Pérez et al. [25]; Pérez-Dueñas et al. [26]
c.256-1G>C	Acceptor, intron 3	Exon 3 and 4 skipping	Pathogenic	Vega et al. [35]
c.256-2A>G	Acceptor, intron 3	No	Pathogenic Likely pathogenic	
c.340-23A>G	Branch site, intron 7	Exon 8 skipping	N/A	Vuillaumier-Barrot et al. [37]
c.347+1G>A	Donor, intron 4	No	Likely pathogenic	
c.348-1G>C	Acceptor, intron 4	No	Likely pathogenic	
c.348-2A>C	Acceptor, intron 4	No	Likely pathogenic	
c.349G>T	Acceptor, intron 4	No	Pathogenic Likely pathogenic	
c.349G>C	Acceptor, intron 4	No	Pathogenic Likely pathogenic	
c.394A>T	Splicing enhancer, exon 5	Exon 5 skipping	N/A	Görlacher et al. [14]; le Bizec et al. [5]
c.447+5G>A	Donor, intron 5	No	Likely pathogenic	
c.447 + 3dupA	Donor, intron 5	Exon 5 skipping	N/A	Slaba et al. [33]
c.448-1_448del	Acceptor, intron 5	No	Likely pathogenic	
c.524-2A>G	Acceptor, intron 6	No	Likely pathogenic	
c.639+1G>A	Donor, intron 7	No	Likely pathogenic	
c.640-9T>G	Acceptor, intron 7	Activation of a cryptic intronic splice-site	Pathogenic Likely pathogenic	Pérez et al. [25]; Richard et al. [28]; Vega et al. [35]
c.640G>A	Acceptor, exon 8	No	Pathogenic VUS	le Bizec et al. [5]; Monin et al. [23]; Schollen et al. [31]; Vicario et al. [36]
c.640-15479C>T	Intron 7	Activation of pseudoexon in intron 7	Likely pathogenic	Liquori et al. [19]; Schollen et al. [32]; Truin et al. [34]; Vega et al. [35]

## 6 Summary

In conclusion, the *PMM2* c.178G>T mutation is a Latino pathogenic variant that causes leaky non-functional alternative splicing inducing frameshift mutations (p.V60Cfs*3). Mutations should be studied concerning their potential disruption of splicing, particularly if they affect canonical splicing sites.

## Author statement

**CAG-D and I-MD:** conceptualization. **CAG-D, MR-M, CE-V, SM-H, RS-M, MEC-M FO-R, AG-J, K-B, CM-G, KC-S, LLF-L, MJ-O, A-MR and CA-V:** investigation. **CAG-D, CA-V and I-MD:** data curation. **CAG-D and IM-D:** writing original draft. **CAG-D, HMM-M, CA-V and IM-D:** review &editing. **IM-D** provided project administration and funding acquisition. **IM-D, CA-V and HMM-M** supervision and validation.

## References

[1] Altassan R., Péanne R., Jaeken J., Barone R., Bidet M., Borgel D., Brasil S., Cassiman D., Cechova A., Coman D., Corral J., Correia J., de la Morena-Barrio M.E., de Lonlay P., dos Reis V., Ferreira C.R., Fiumara A., Francisco R., Freeze H., Morava E. (2019). International clinical guidelines for the management of phosphomannomutase 2-congenital disorders of glycosylation: diagnosis, treatment and follow up. J. Inherit. Metab. Dis..

[2] Andreotti G., de Vaca I.C., Poziello A., Monti M.C., Guallar V., Cubellis M.V. (2014). Conformational response to ligand binding in Phosphomannomutase2: insights into inborn glycosylation disorder. J. Biol. Chem..

[3] Asteggiano C.G., Papazoglu M., Bistué Millón M.B., Peralta M.F., Azar N.B., Spécola N.S., Guelbert N., Suldrup N.S., Pereyra M., Dodelson de Kremer R. (2018). Ten years of screening for congenital disorders of glycosylation in Argentina: case studies and pitfalls. Pediatr. Res..

[4] Bahena-Bahena D., López-Valdez J., Raymond K., Salinas-Marín R., Ortega-García A., Ng B.G., Freeze H.H., Ruíz-García M., Martínez-Duncker I. (2014). ATP6V0A2 Mutations Present in Two Mexican Mestizo children With an Autosomal Recessive Cutis Laxa Syndrome Type IIA. http://europepmc.org/abstract/med/27896089.

[5] le Bizec C., Vuillaumier-Barrot S., Barnier A., Dupré T., Durand G., Seta N. (2005). A new insight into PMM2 mutations in the French population. Hum. Mutat..

[6] Briones P., Vilaseca M.A., Schollen E., Ferrer I., Maties M., Busquets C., Artuch R., Gort L., Marco M., van Schaftingen E., Matthijs G., Jaeken J., Chabás A. (2002). Biochemical and molecular studies in 26 Spanish patients with congenital disorder of glycosylation type Ia. J. Inherit. Metab. Dis..

[7] Brum J.M. (2013). Clinical and molecular features of patients with congenital disorders of glycosylation in Brazil. Pediat. Therap..

[8] Cabezas O.R., Flanagan S.E., Stanescu H., García-Martínez E., Caswell R., Lango-Allen H., Antón-Gamero M., Argente J., Bussell A.M., Brandli A., Cheshire C., Crowne E., Dumitriu S., Drynda R., Hamilton-Shield J.P., Hayes W., Hofherr A., Iancu D., Issler N., Bockenhauer D. (2017). Polycystic kidney disease with hyperinsulinemic hypoglycemia caused by a promoter mutation in phosphomannomutase 2. J. Am. Soc. Nephrol..

[9] Desmet F.O., Hamroun D., Lalande M., Collod-Bëroud G., Claustres M., Béroud C. (2009). Human splicing finder: an online bioinformatics tool to predict splicing signals. Nucleic Acids Res..

[10] Freeze, H. H. (2019). Improving biochemical markers for disorders of N-glycosylation. Ann. Transl. Med., 7(S6). Doi:10.21037/atm.2019.07.79. 10.21037/atm.2019.07.79 PMC6789335 31656755

[11] Freeze H.H., Hart G.W., Schnaar R.L. (2017). Glycosylation precursors.

[12] García-Moreno J.F., Romão L. (2020). Perspective in alternative splicing coupled to nonsense-mediated mrna decay. Int. J. Mol. Sci..

[13] González-Domínguez C.A., Raya-Trigueros A., Manrique-Hernández S., González Jaimes A., Salinas-Marín R., Molina-Garay C., Carrillo-Sánchez K., Flores-Lagunes L.L., Jiménez-Olivares M., Dehesa-Caballero C., Alaez-Versón C., Martínez-Duncker I. (2020). Identification through exome sequencing of the first PMM2-CDG individual of Mexican mestizo origin. Mol. Genet. Metabol. Reports.

[14] Görlacher M., Panagiotou E., Himmelreich N., Hüllen A., Beedgen L., Dimitrov B., Geiger V., Zielonka M., Peters V., Strahl S., Vázquez-Jiménez J., Kerst G., Thiel C. (2020). Fatal outcome after heart surgery in PMM2-CDG due to a rare homozygous gene variant with double effects. Mol. Genet. Metabol. Reports.

[15] Grünewald S., Schollen E., van Schaftingen E., Jaeken J., Matthijs G. (2001). High residual activity of PMM2 in patients’ fibroblasts: possible pitfall in the diagnosis of CDG-Ia (phosphomannomutase deficiency). Am. J. Hum. Genet..

[16] Hernandez-Nieto C., Alkon-Meadows T., Lee J., Cacchione T., Iyune-Cojab E., Garza-Galvan M., Luna-Rojas M., Copperman A.B., Sandler B. (2020). Expanded carrier screening for preconception reproductive risk assessment: prevalence of carrier status in a Mexican population. Prenat. Diagn..

[17] Jones M.A., Rhodenizer D., da Silva C., Huff I.J., Keong L., Bean L.J.H., Coffee B., Collins C., Tanner A.K., He M., Hegde M.R. (2013). Molecular diagnostic testing for congenital disorders of glycosylation (CDG): detection rate for single gene testing and next generation sequencing panel testing. Mol. Genet. Metab..

[18] Karczewski K.J., Francioli L.C., Tiao G., Cummings B.B., Alföldi J., Wang Q., Collins R.L., Laricchia K.M., Ganna A., Birnbaum D.P., Gauthier L.D., Brand H., Solomonson M., Watts N.A., Rhodes D., Singer-Berk M., England E.M., Seaby E.G., Kosmicki J.A., MacArthur D.G. (2020). The mutational constraint spectrum quantified from variation in 141,456 humans. Nature.

[19] Liquori A., Vaché C., Baux D., Blanchet C., Hamel C., Malcolm S., Koenig M., Claustres M., Roux A.F. (2016). Whole USH2A gene sequencing identifies several new deep intronic mutations. Hum. Mutat..

[20] de Magalhães A.P.P.S., Burin M.G., de Souza C.F.M., de Bitencourt F.H., Sebastião F.M., Silva T.O., Vairo, F. P. e., & Schwartz, I. V. D. (2020). Transferrin isoelectric focusing for the investigation of congenital disorders of glycosylation: analysis of a ten-year experience in a Brazilian center. J. Pediatr..

[21] Marklová E., Albahri Z. (2007). Screening and diagnosis of congenital disorders of glycosylation. Clin. Chim. Acta.

[22] Matthijs G., Schollen E., Bjursell C., Erlandson A., Freeze H., Imtiaz F., Kjaergaard S., Martinsson T., Schwartz M., Seta N., Vuillaumier-Barrot S., Westphal V., Winchester B. (2000). Mutations in PMM2 that cause congenital disorders of glycosylation, type Ia (CDG-Ia). Hum. Mutat..

[23] Monin M.L., Mignot C., de Lonlay P., Héron B., Masurel A., Mathieu-Dramard M., Lenaerts C., Thauvin C., Gérard M., Roze E., Jacquette A., Charles P., de Baracé C., Drouin-Garraud V., Khau Van Kien P., Cormier-Daire V., Mayer M., Ogier H., Brice A., Héron D. (2014). 29 French adult patients with PMM2-congenital disorder of glycosylation: outcome of the classical pediatric phenotype and depiction of a late-onset phenotype. Orphanet J. Rare Dis..

[24] Ondruskova N., Cechova A., Hansikova H., Honzik T., Jaeken J. (2021). Congenital disorders of glycosylation: still “hot” in 2020. Biochim. Biophys. Acta Gen. Subj..

[25] Pérez B., Vega A.I., Ecay M.J., Ugarte M., Pérez-Cerda C., Pérez B., Vega A.I., Ecay M.J., Ugarte M., Pérez-Cerda C., Briones P., Briones P., Briones P., Quelhas D., Artuch R., Artuch R., Quintana E., Gort L., Quintana E., Matthijs G. (2011). The molecular landscape of phosphomannose mutase deficiency in iberian peninsula: identification of 15 population-specific mutations. J. Inherit. Metab. Dis..

[26] Pérez-Dueñas B., García-Cazorla A., Pineda M., Poo P., Campistol J., Cusí V., Schollen E., Matthijs G., Grunewald S., Briones P., Pérez-Cerdá C., Artuch R., Vilaseca M.A. (2009). Long-term evolution of eight Spanish patients with CDG type Ia: typical and atypical manifestations. Eur. J. Paediatr. Neurol..

[27] Pirard M., Matthijs G., Heykants L., Schollen E., Grünewald S., Jaeken J., van Schaftingen E. (1999). Effect of mutations found in carbohydrate-deficient glycoprotein syndrome type IA on the activity of phosphomannomutase 2. FEBS Lett..

[28] Richard E., Vega A.I., Pérez B., Roche C., Velázquez R., Ugarte M., Pérez-Cerdá C. (2009). Congenital disorder of glycosylation Ia: new differentially expressed proteins identified by 2-DE. Biochem. Biophys. Res. Commun..

[29] Richards S., Aziz N., Bale S., Bick D., Das S., Gastier-Foster J., Grody W.W., Hegde M., Lyon E., Spector E., Voelkerding K., Rehm H.L. (2015). Standards and guidelines for the interpretation of sequence variants: a joint consensus recommendation of the American College of Medical Genetics and Genomics and the Association for Molecular Pathology. Genet. Med..

[30] Schollen E., Kjaergaard S., Legius E., Schwartz M., Matthijs G. (2000). Lack of hardy-Weinberg equilibrium for the most prevalent PMM2 mutation in CDG-Ia (congenital disorders of glycosylation type Ia). Eur. J. Hum. Genet..

[31] Schollen E., Martens K., Geuzens E., Matthijs G. (2002). DHPLC analysis as a platform for molecular diagnosis of congenital disorders of glycosylation (CDG). Eur. J. Hum. Genet..

[32] Schollen E., Keldermans L., Foulquier F., Briones P., Chabas A., Sánchez-Valverde F., Adamowicz M., Pronicka E., Wevers R., Matthijs G. (2007). Characterization of two unusual truncating PMM2 mutations in two CDG-Ia patients. Mol. Genet. Metab..

[33] Slaba K., Noskova H., Vesela P., Tuckova J., Jicinska H., Honzik T., Hansikova H., Kleiblova P., Stourac P., Jabandziev P., Slaby O., Prochazkova D. (2020). Novel splicing variant in the PMM2 gene in a patient with PMM2-CDG syndrome presenting with pericardial effusion: a case report. Front. Genet..

[34] Truin G., Guillard M., Lefeber D.J., Sykut-Cegielska J., Adamowicz M., Hoppenreijs E., Sengers R.C.A., Wevers R.A., Morava E. (2008). Pericardial and abdominal fluid accumulation in congenital disorder of glycosylation type Ia. Mol. Genet. Metab..

[35] Vega A.I., Perez-Cerda C., Desviat L.R., Matthijs G., Ugarte M., Pérez B. (2009). Functional analysis of three splicing mutations identified in the PMM2 gene: toward a new therapy for congenital disorder of glycosylation type IA. Hum. Mutat..

[36] Vicario M., Calì T., Cieri D., Vallese F., Bortolotto R., Lopreiato R., Zonta F., Nardella M., Micalizzi A., Lefeber D.J., Valente E.M., Bertini E., Zanotti G., Zanni G., Brini M., Carafoli E. (2017). A novel PMCA3 mutation in an ataxic patient with hypomorphic phosphomannomutase 2 (PMM2) heterozygote mutations: biochemical characterization of the pump defect. Biochim. Biophys. Acta Mol. basis Dis..

[37] Vuillaumier-Barrot S., le Bizec C., de Lonlay P., Madinier-Chappat N., Barnier A., Dupré T., Durand G., Seta N. (2006). PMM2 intronic branch-site mutations in CDG-Ia. Mol. Genet. Metab..

